# Chlorin e6-Biotin Conjugates for Tumor-Targeting Photodynamic Therapy

**DOI:** 10.3390/molecules26237342

**Published:** 2021-12-03

**Authors:** Wei Liu, Xingqun Ma, Yingying Jin, Jie Zhang, Yang Li, Yuxia Tang, Yong Song, Shouju Wang

**Affiliations:** 1 Lab of Molecular Imaging, Department of Radiology, the First Affiliated Hospital of Nanjing Medical University, Nanjing 210000, China; njmu_lw@163.com (W.L.); yingyingjin@njmu.edu.cn (Y.J.); zhangjiegxf@163.com (J.Z.); yang.li@njmu.edu.cn (Y.L.); 2 Department of Oncology, Nanjing Baiyi Hospital, Jinling Clinical College of Nanjing Medical University, Nanjing 210000, China; maxingqun@126.com; 3 Department of Respiratory and Critical Care Medicine, Jinling Clinical College of Nanjing Medical University, Nanjing 210000, China

**Keywords:** biotin, photosensitizer, chlorin e6, photodynamic therapy

## Abstract

To improve the tumor-targeting efficacy of photodynamic therapy, biotin was conjugated with chlorin e6 to develop a new tumor-targeting photosensitizer, Ce6-biotin. The Ce6-biotin had good water solubility and low aggregation. The singlet-oxygen generation rate of Ce6-biotin was slightly increased compared to Ce6. Flow cytometry and confocal laser scanning microscopy results confirmed Ce6-biotin had higher binding affinity toward biotin-receptor-positive HeLa human cervical carcinoma cells than its precursor, Ce6. Due to the BR-targeting ability of Ce6-biotin, it exhibited stronger cytotoxicity to HeLa cells upon laser irradiation. The IC50 against HeLa cells of Ce6-biotin and Ce6 were 1.28 µM and 2.31 µM, respectively. Furthermore, both Ce6-biotin and Ce6 showed minimal dark toxicity. The selectively enhanced therapeutic efficacy and low dark toxicity suggest that Ce6-biotin is a promising PS for BR-positive-tumor-targeting photodynamic therapy.

## 1. Introduction

Photodynamic therapy (PDT) is based on the local activation of photosensitizers (PSs) in tumors to induce chemical damage, leading to tumor cell death [1,2]. This method has become a clinically promising approach for treatment of cancers and a wide range of other diseases. Upon irradiation, the PSs activate oxygen to the singlet state and damage the nearby cells [3,4,5]. Compared with ultraviolet or visible light, near-infrared (NIR) light has deeper penetration into the normal tissues and has great potential in PDT for deep tumors [6]. However, PSs may accumulate in healthy tissues and cause various side effects, such as pain and photoallergic reactions [4,7]. Up to now, the poor selectivity of PSs to tumors is still an obstacle that severely limits their clinical application in PDT [8,9]. Therefore, much research has been devoted to improving the targeting ability of PSs.

The targeting efficacy of PDT is contingent on the relative abundance of PSs in tumor lesions compared to the surrounding normal tissues [10]. Selective toxicity would be achieved if PSs were delivered to specific membrane receptors of target cells with the aid of complementary targeting moieties (antibodies, saccharides, hormones, lectins). By illuminating the target area only, dual-specificity would be achieved by the specific distribution of PSs and the spatial control of the irradiation field [10,11]. Combining targeting ligands with PSs is a promising strategy to achieve tumor-specific distribution. Biotin (vitamin B7) is one of the excellent targeting ligands to target cancer cells. Various cancer cells, such as cervical, breast, lung, and ovarian, overexpress biotin receptors [12,13,14]. Thus, biotin has been combined with various fluorescent materials to improve the efficiency of cell labeling and bioimaging [15]. Nilesh et al. [14] designed biotin-tagged nanocarriers for drug delivery. Their results showed significantly higher accumulation of nanocarriers in biotin-receptor-overexpressed cervical cancer cells (HeLa) through the receptor-assisted endocytosis process, which significantly enhanced the therapeutic effect on cancer cells. Moreover, the chemical structure of the biotin molecule is composed of two rings and a valeric acid moiety attached as a side chain. The valeric acid moiety enables biotin to participate in various syntheses [16]. A few PSs, like methyl-pheophorbide and phthalocyanine, have been conjugated to biotin and showed improved therapeutic efficacy to biotin-receptor- (BR) positive cancer cells [17,18].

One of the most widely used PSs is Chlorin e6 (Ce6). Ce6 has the following advantages: rapid clearance [17], high singlet-oxygen-generation efficiency [19], NIR light excitation [20], and fluorescence imaging capability [21]. Ce6 is currently under clinical trials for the treatment of esophagus, bladder, skin, head, and neck cancers [22]. Ce6 and its conjugates are minimally toxic in the dark; however, the biodistribution of Ce6 in the body is non-specific and may induce photosensitivity reactions when patients are exposed to sunlight. Therefore, the targeted delivery of Ce6 is highly desirable.

In this work, we conjugated biotin with Ce6 to synthesize tumor-targeting PSs and to increase its therapeutic efficacy to BR-positive cancer cells. The water solubility and singlet-oxygen-generating ability of Ce6-biotin were measured. The targeting ability, PDT activities and dark toxicity of Ce6-biotin were tested in a BR-positive cell line (HeLa cell). The experimental results show that Ce6-biotin is a potential tumor-targeting PS for PDT.

## 2. Results and Discussion

### 2.1. Synthesis of Ce6-Biotin

**Scheme 1:**
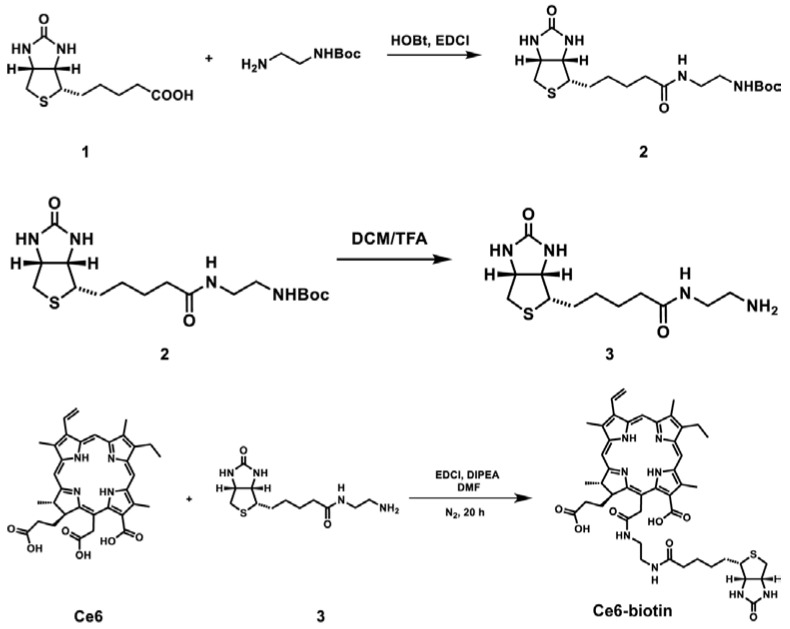
Synthesis of Ce6-biotin.

Scheme 1 shows the synthetic route of Ce6-biotin. A Boc-protected amino group was linked to the biotin molecule to obtain compound **2** through Ethyl-3-(3-dimethylaminopropyl) carbodiimide hydrochloride (EDCI) reaction. Then, the amino group was de-protected (compound **3**) and linked to the carboxyl group on Ce6 to obtain Ce6-biotin. The reaction is selective to the middle carboxyl group due to its high reactivity [23]. The ^1^H-NMR of Boc-protected biotin and Ce6-biotin and the HR-MS of Ce6-biotin are available in Supplementary Material (Figures S1–S3).

### 2.2. Photophysical Properties of Ce6-Biotin

**Figure 1:**
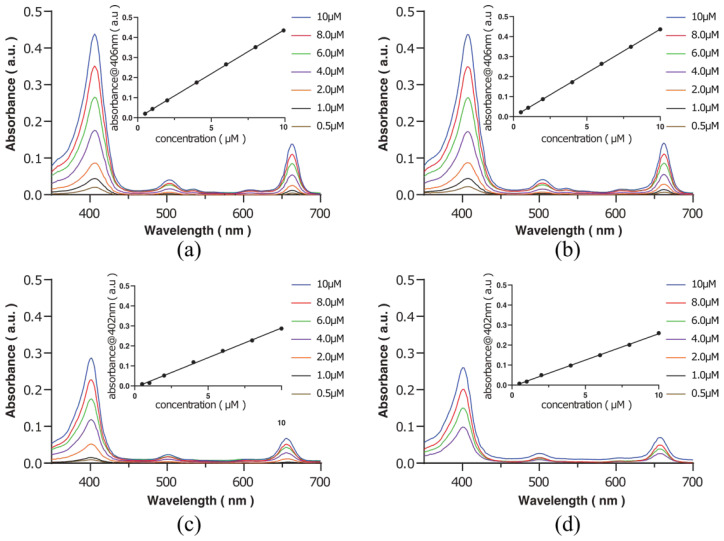
UV-Vis spectra of Ce6 in DMSO (**a**) and PBS (**c**), as well as Ce6-biotin in DMSO (**b**) and PBS (**d**). The insets plot the absorbance of the Q-band versus the concentration of Ce6 and Ce6-biotin.

To evaluate the photophysical properties of Ce6-biotin, ultraviolet-visible spectroscopy (UV-vis spectra) of Ce6-biotin was investigated both in phosphate buffer saline (PBS) and dimethyl sulfoxide (DMSO). The UV–vis spectra of Ce6 and Ce6-biotin at different concentrations are shown in Figure 1. The Q-bands of both Ce6 and Ce6-biotin strictly obey the Lambert-Beer law. The UV-vis spectra in DMSO and PBS indicate a typical absorption of non-aggregated Ce6, displaying a narrow and strong Soret band at 406 nm in DMSO and 402 nm in PBS, respectively, along with a Q-band at 655 nm. The absorption spectra in PBS are similar to those in DMSO, which has just a 4 nm red shift of the Q-band. As aggregation is concentration-dependent, the relationship between the UV-vis spectra and the concentrations of Ce6-biotin was also investigated. No new bands appear, with the concentration varying from 0.5 to 10 μM in PBS, further proving that Ce6-biotin is non-aggregated in PBS. The lower absorbance of Ce6 and Ce6-biotin in PBS is probably due to the solute-solvent interaction [24]. The non-aggregated state of PSs is very important for their PDT efficiency because aggregation of PSs may inhibit the generation efficiencies of ROSs [10,25,26].

**Figure 2:**
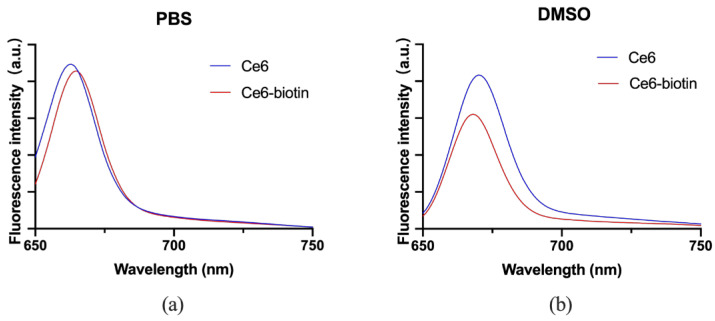
Fluorescence spectra of Ce6 and Ce6-biotin (2 μM) in PBS (**a**) and DMSO (**b**).

The fluorescence of Ce6 and Ce6-biotin in PBS was compared. Upon excitation at 400 nm, Ce6 shows fluorescence emission peaks at 663 nm in PBS and 670 nm in DMSO, while Ce6-biotin shows fluorescence emission peaks at 665 nm in PBS and 668 in DMSO. The absorption spectrum and the fluorescence intensity remained virtually the same (Figure 2). The fluorescence of Ce6-biotin is only a little weaker than that of Ce6, probably because the modification did not alter the basic structure of Ce6. Considering its intense fluorescence emission in the infrared region, Ce6-biotin may also serve as a fluorescent probe for in vivo application [27].

### 2.3. Detecting Singlet-Oxygen Generation In Vitro

**Figure 3:**
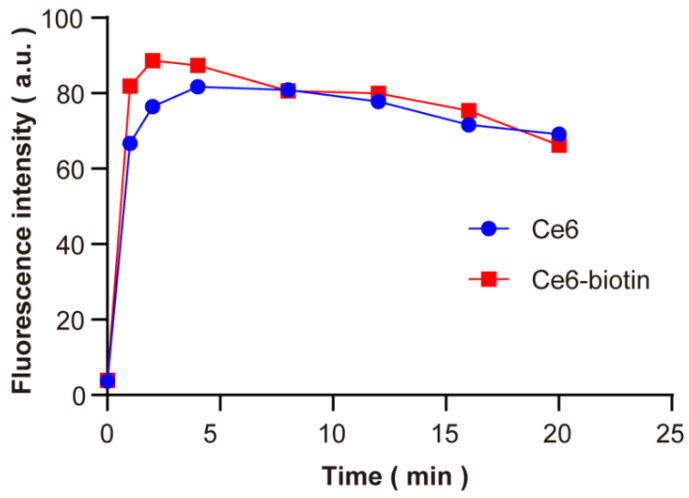
Comparison of the fluorescence of SOSG at 530 nm in PBS using Ce6 and Ce6-biotin as photosensitizers after being irradiated by 660 nm (1 W·cm^−2^) laser up to 20 min.

The singlet-oxygen-generating efficiency of Ce6-biotin was measured by the Singlet Oxygen Sensor Green (SOSG) assay. Upon the presence of ^1^O_2_, the intrinsic fluorescence of SOSG in PBS is restored, resulting in increased fluorescence with laser irradiation time. As shown in Figure 3, Ce6-biotin was an efficient singlet-oxygen generator, and the efficiency was similar to Ce6. It shows that the introduction of biotin will not affect the ability of Ce6 to produce singlet oxygen. The PDT efficiency of PSs is highly dependent on their singlet-oxygen generation abilities [28,29]. As shown in Table 1, the ^1^O_2_ quantum yield of Ce6-biotin (Φ_Δ_ = 0.81) is slightly higher than that of Ce6 (Φ_Δ_ = 0.75), which might be attributed to the large conjugation of the porphyrinic system [10,30]. All these results clearly indicate the singlet-oxygen generation ability of Ce6-biotin.

### 2.4. Cellular Uptake of Ce6-Biotin

**Figure 4:**
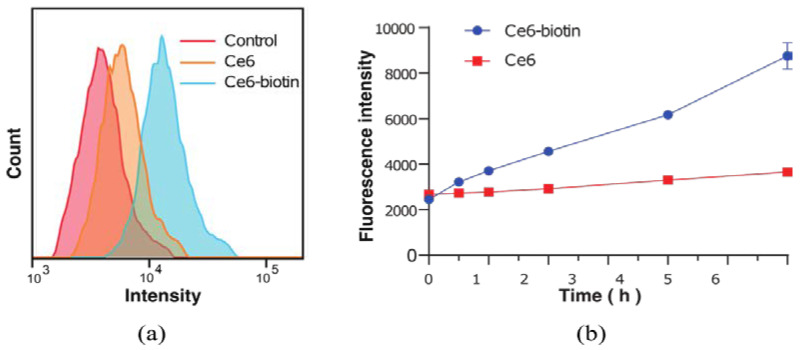
(**a**) Flow cytometry analyses of HeLa cells incubated with Ce6 and Ce6-biotin for 6 h. (**b**) Uptake isotherms of Ce6 and Ce6-biotin toward HeLa cells incubated for 0.5, 1, 2, 4, and 6 h, respectively.

The cellular uptake of Ce6 and Ce6-biotin in HeLa cells was investigated by a flow-cytometry collection method since biotin receptors are overexpressed in HeLa cells. PSs (2 μM) were incubated with HeLa cells for different times (0.5, 1, 2, 4 and 6 h). After incubation, the internalized PSs were quantified by flow cytometer. As shown in Figure 4a, the uptake of Ce6-biotin in HeLa cells is significantly higher than that of Ce6 for 6 h. After quantitative analysis (Figure 4b), the cellular uptake of Ce6-biotin after 6 h incubation is about 4 times higher than that of Ce6. The rapid differentiation of Ce6 and Ce6-biotin in cellular uptake supports the assumption that conjugation of Ce6 with biotin molecules can enhance the association of Ce6 toward BR-positive HeLa cells.

**Figure 5:**
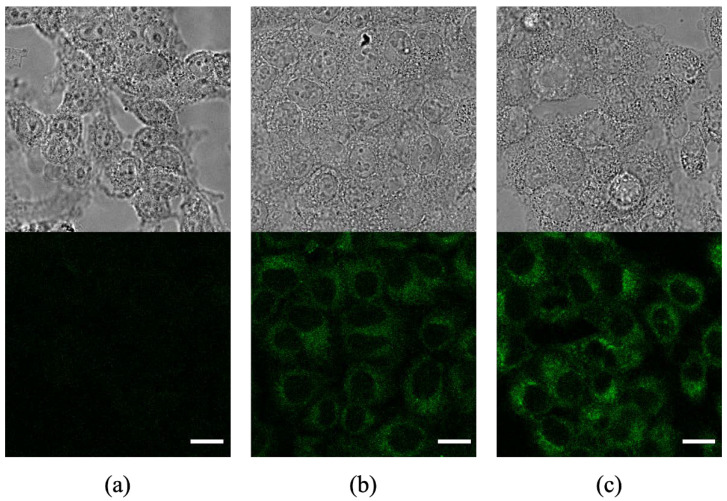
Confocal fluorescence images of HeLa cells without incubation with photosensitizers (**a**) and after incubation with Ce6 (**b**) and Ce6-biotin (**c**). Scale bars: 20 µm.

The cellular uptake of Ce6-biotin was also visualized by confocal fluorescence imaging. The fluorescence images were obtained for both Ce6- and Ce6-biotin-treated cells after 6 h (Figure 5). It is shown that both Ce6 and Ce6-biotin were internalized by HeLa cells. It is noted that the intracellular fluorescence intensity of Ce6-biotin was higher than that of Ce6, which is in line with the observed higher uptake of Ce6-biotin than Ce6 in HeLa cells.

### 2.5. Therapeutic Efficacy and Dark Cytotoxicity of Ce6-Biotin

**Figure 6:**
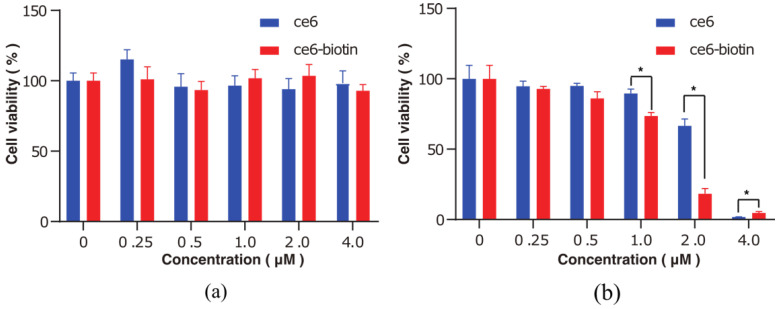
Cytotoxicity against HeLa cells of Ce6 and Ce6-biotin in the dark (**a**) or upon irradiation ((**b**), λ = 655 nm). *p*-values < 0.05 are denoted with asterisks (*).

**Figure 7:**
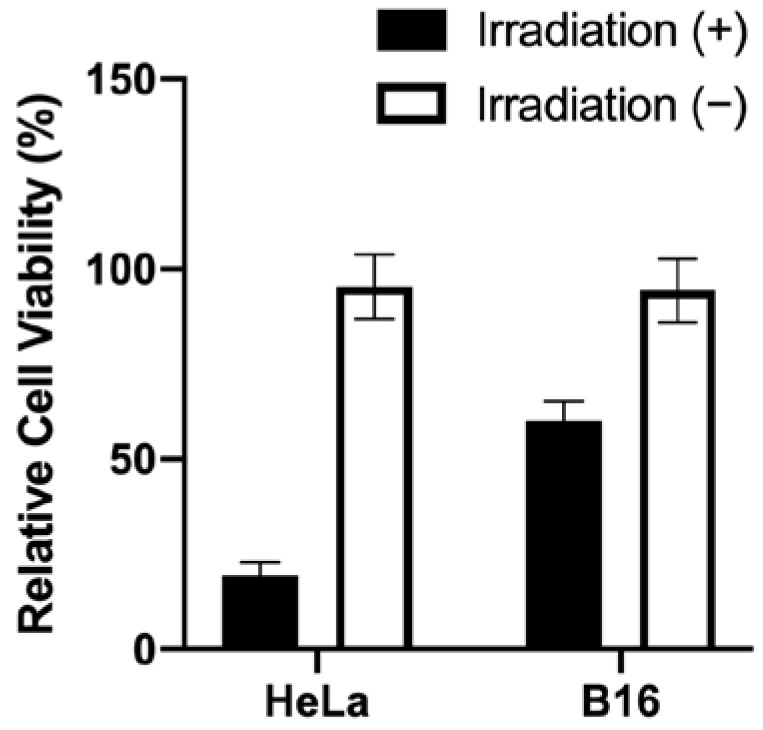
Cytotoxicity against HeLa (BR+) and B16 (BR−) cells with and without irradiation (20 J·cm^−2^) after incubation with 2.0 µM Ce6-biotin for 24 h.

In order to measure the therapeutic efficacy and dark cytotoxicity of Ce6 and Ce6-biotin, the 3-(4,5-dimethylthiazol-2-yl)-2,5-diphe-yltetrazolium bromide (MTT) assay was used to determine the viability of the cells treated with the two PSs. The cells treated at zero concentration were set as control groups. Without irradiation, Ce6 and Ce6-biotin showed minimal cytotoxicity to HeLa cells at concentrations between 0.25 and 4 μM (Figure 6a). After irradiation, both Ce6 and Ce6-biotin exhibited concentration-dependent cytotoxicity. The assays showed the viability of HeLa cells decreased from 100 ± 9.57% of the blank control to 1.83 ± 0.08% (Ce6) and 4.72 ± 1.03% (Ce6-biotin) after laser irradiation, as the Ce6 and Ce6-biotin concentration increased from 0.25 to 4.0 µM (Figure 6b). It is noted that at concentrations from 1.0 μM to 2.0 μM, the therapeutic efficacy of Ce6-biotin is significantly higher than that of Ce6 at the same concentration. At concentrations of 4.0 μM, the therapeutic efficacy of Ce6 is significantly higher than that of Ce6-biotin. This is in line with the observed higher uptake of Ce6-biotin than Ce6 in HeLa cells. The calculated IC 50 of Ce6 is 2.31 μM—higher than that of Ce6-biotin (1.28 μM). Furthermore, the cytotoxicity of Ce6-biotin was tested in HeLa (BR+) and B16 (BR−) cells both with and without irradiation. As shown in Figure 7, Ce6-biotin exhibited higher cytotoxicity to HeLa cell than to B16 cells after treatment with the same light dose (20 J·cm^−2^) and Ce6-biotin concentration (2.0 µM). These results indicate that the Ce6-biotin can enhance the therapeutic effect on BR-positive cancer cells without increasing dark cytotoxicity.

## 3. Materials and Methods

### 3.1. Materials

Chlorin e6, biotin, *N*-Hydroxybenzotrizole (HOBt), EDCI, *N*,*N*-Dimethylformamide (DMF), dichloromethane (DCM), trifluoroacetic acid (TFA), and 4-piperidinol,*N*,*N*-diisopropylethylamine (DIPEA) were obtained from Sigma-Aldrich (St. Louis, MO, USA). SOSG was purchased from Thermo Fisher Scientific, Inc. (Waltham, MA, USA). HeLa cells were purchased from the Type Culture Collection of the Chinese Academy of Sciences, Shanghai, China. High-glucose Dulbecco’s modified Eagle medium (DMEM), Trypsin-EDTA (0.25%), heat-inactivated fetal bovine serum (FBS), and penicillin-streptomycin solution were purchased from Thermo Fisher Scientific, Inc. (Waltham, MA, USA).

### 3.2. Synthesis of Ce6-Biotin

Biotin as compound **1** (121.5 mg, 0.5 mmol), HOBt (80.65 mg, 0.6 mmol) and EDCI (114 mg, 0.6 mmol) were stirred in anhydrous DMF under ice-cold conditions for 15 min. Mono-Boc-protected ethylene diamine (0.5 mmol) dissolved in anhydrous DMF was slowly added. The corresponding reaction mixture was allowed to stir for 12 h at room temperature. When the reaction was finished, water (100 mL) was added, and the aqueous layer was extracted with DCM (3 × 10 mL). The combined organic layer was dried over anhydrous Na_2_SO_4_ and concentrated under vacuum. The crude product obtained was purified by silica gel chromatography (with 6% MeOH in DCM) to afford compound **2** as a white solid. Yield: 67.5 mg, (35%). ^1^H-NMR (300 MHz, CD 3 OH) δ 4.48–4.33 (m, 1H), 4.30–4.14 (m, 1H), 3.30–2.98 (m, 7H), 2.82 (dd, *J* = 12.6, 4.8 Hz, 1H), 2.60 (d, *J* = 12.5 Hz, 1H), 2.11 (d, *J* = 7.2 Hz, 2H), 1.74–1.46 (m, 4H), 1.33 (s, 9H). Compound **2** was then dissolved in DCM/TFA (3:1) and stirred for 3 h at room temperature to obtain compound **3**. The mixture was concentrated under vacuum.

The obtained compound **3** was reacted with Ce6 without further purification. Then, Ce6 (10 mg, 1 eq.) was dissolved in DMF. EDCI (1.5 eq.), and compound **3** (1.4 eq.) and DIPEA were added to the reaction mixture. The mixture was stirred overnight at room temperature. The reaction was monitored by TLC. The reaction mixture was diluted with water and adjusted to pH 3 with 5% citric acid aqueous solution and extracted with CH_2_Cl_2_ (three times). The CH_2_Cl_2_ layer was washed with brine and dried over anhydrous Na_2_SO_4_. Removal of the solvent under reduced pressure gave a crude product. The crude product was purified by prep-HPLC on an X Bridge C18 chromatographic column using a linear gradient from 30% to 40% acetonitrile in MeCN/H_2_O (0.1% NH_4_HCO_3_) to obtain Ce6-biotin. Yield: 2 mg, (13.8%). ^1^H-NMR (400 MHz, DMSO) δ 9.78 (s, 1H), 9.66 (s, 1H), 9.12 (s, 1H), 9.02 (s, 1H), 8.35 (dd, *J* = 17.8, 11.7 Hz, 1H), 6.43 (d, *J* = 18.2 Hz, 1H), 6.13 (d, *J* = 12.0 Hz, 1H), 6.09 (s, 1H), 5.98 (s, br, 1H), 4.71 (d, *J* = 6.3 Hz, 1H), 4.56 (d, *J* = 6.8 Hz, 1H), 3.81 (d, *J* = 7.0 Hz, 2H), 3.53 (s, 3H), 3.47 (s, 3H), 3.05 (s, br, 4H), 2.23 (s, br, 2H), 1.95 (s, br, 3H), 1.66 (t, *J* = 7.5 Hz, 3H), 1.60 (d, *J* = 6.4 Hz, 2H), 1.32 (s, 1H), 1.20 (s, 3H), 0.95 (s, br, 3H). HR-MS found: *m/z*: 865.4067 [M + H]^+^,calcd for: C_46_H_57_N_8_O_7_S. The purity was found to be ~90% by HPLC analysis.

### 3.3. Photophysical Measurement

Absorption and fluorescence emission spectra were recorded by a UV-Vis spectrophotometer (Lambda 35, PerkinElmer Instruments, Waltham, MA, USA) and spectrofluorometer (F96, Lengguang Tech, Shanghai, China). For fluorescence measurements, the excitation wavelength was set at 400 nm.

### 3.4. Singlet-Oxygen Generation Measurement

First, Ce6 and Ce6-biotin (2 µM, 990 µL) and SOSG (0.5 mM, 10 µL) were mixed in 1.5 mL centrifuge tubes. The final concentration of SOSG was 5µM. After irradiation (660 nm, 1 W·cm^−2^) for different time periods (0, 1, 2, 4, 8, 12, 16, 20 min), the fluorescence intensity was detected using a fluorescence spectrophotometer (F-4600, Hitachi, Tokyo, Japan).

Singlet-oxygen generation yield of Ce6-biotin (2 µM) was evaluated by using SOSG as a probe molecule. Ce6 (ΦΔ = 0.75 in PBS) was employed as a reference PS for singlet-oxygen quantum-yield calculations [31]. For each PS, the slope of emission maxima of SOSG at 530 nm versus the time graph was drawn. Finally, singlet-oxygen quantum yields were calculated according to the Equation (1) given below:

(1) $$\Phi_{\Delta }\;(\mathrm{PS})=\Phi_{\Delta }\;(\mathrm{ref})\;\times \;\;\frac{m\left(\mathrm{PS}\right)}{m\left(\mathrm{ref}\right)}\;\times \;\;\frac{F\left(\mathrm{ref}\right)}{F\left(\mathrm{PS}\right)}\;\times \;\;\frac{\mathrm{PF}\left(\mathrm{ref}\right)}{\mathrm{PF}\left(\mathrm{PS}\right)}$$

where PS represents Ce6-biotin; ref represents Ce6; m is the slope of emission maxima of SOSG at 530 nm versus time graph; F is the correction factor, which is given by F = 1–10 ^−OD|^ (OD, optical density, at the irradiation wavelength, which is 660 nm); and PF is absorbed photonic flux in μEinstein dm^−3^·s^−1^. PF was ignored in the calculations, as both Ce6-biotin and Ce6 were irradiated with the same light source (660 nm, 1 W·cm^−2^).

### 3.5. Cellular Uptake

HeLa cells were maintained in high-glucose DMEM with 10% FBS at 37 °C under a humidified 5% CO_2_ atmosphere. HeLa cells (3 × 10^5^) were seeded in 12-well plates and incubated to confluence. Then, the cells were incubated with the Ce6 and Ce6-biotin (2 μM) for 0.5, 1, 2, 4, and 6 h. The medium was carefully aspirated, then the cells were rinsed 3 times with PBS, trypsinized and collected by centrifugation, and then fixed with 4% paraformaldehyde for 30 min. Cellular uptake was quantitatively measured with a BD LSRFortessa flow cytometer.

### 3.6. Cytotoxicity

MTT assay was utilized to evaluate the cytotoxicity of the Ce6 and Ce6-biotin against the HeLa cells. HeLa cells were seeded at 6 × 10^3^ per/well in costar 96-well plates and incubated for 12 h to confluence. The cells were treated with various concentrations (0.01–4 μM) of the Ce6 and Ce6-biotin for 24 h at 37 °C. Then, the cells were washed with PBS and refilled with culture medium. The cells were irradiated with an LED assay (λ ≈ 655 nm, 6.0 mW·cm^2^) for 60 min, and the total light dose was 20 J·cm^−2^. After 24 h incubation, MTT solution was added to each well, and the cells were incubated for another 4 h. Then the MTT solutions were removed, and the wells were refilled with 100 μL of DMSO. The cell viability date was obtained by measuring the absorbance at 570 nm and reference at 630 nm of each well using a Tecan i-control infinite 200Pro. Dark controls were measured using the above method without illumination.

### 3.7. Intracellular Fluorescence Images

For confocal laser scanning microscopy, HeLa cells were seeded in a Lab-Tek chamber slide system (Thermo Fisher Scientific, Waltham, MA, USA) with 5 × 10^3^ cells per well in DMEM (200 μL) containing 10% FBS and cultured in a 1% O_2_ /5% CO_2_ humidified atmosphere for 24 h at 37 °C. Then, cells were incubated with Ce6 and Ce6-biotin for 6 h. After the cells were washed with PBS several times, they were stained with UltraCruz mounting medium (Santa Cruz Biotechnology, Dallas, TX, USA). Cellular uptake of Ce6 was measured using confocal laser scanning microscopy (STELLARIS Confocal Microscope, Leica, Germany).

## 4. Conclusions

Herein, we synthesized and characterized a new BR-targeted Ce6 compound for selective delivery of PSs to cancer cells that overexpressed the receptors of these ligands. Ce6-biotin exhibits highly efficient singlet-oxygen generation, which is similar to Ce6. For the BR-targeted PS, we detected an increase in its internalization in HeLa cells, indicating that Ce6-biotin can selectively accumulate in the BR-positive HeLa human cervical carcinoma cells, resulting in higher cellular uptake than its precursor, Ce6. Moreover, under irradiation with a red laser, Ce6-biotin exhibited stronger antitumor efficacy, combined with minimal dark toxicity. These findings demonstrate that Ce6-biotin is a promising PS for BR-positive-tumor-targeting PDT therapy and worthy of further investigation.

## Figures and Tables

**Table 1:** Photochemical data for Ce6 and Ce6-biotin.

	λ_abs_ (nm)	λ_em_ (nm) ^a^	Φ_Δ_ ^b^
Ce6	406	663	0.75
Ce6-biotin	402	665	0.81

^a^ Excited at 400 nm. ^b^ Referenced by Ce6 (Φ_Δ_ = 0.75 in PBS).

## Data Availability

The data used to support the findings of this study are available from the corresponding author upon request.

## Supplementary Materials

The following are available online: Figure S1: The ^1^H-NMR of Boc-protected biotin (compound **2**), Figure S2: The ^1^H-NMR of Ce6-biotin, Figure S3: The HR-MS of Ce6-biotin.

## Sample Availability

Samples of the compound, Ce6-biotin is available from the authors.
