# Supplementary material for: Chlorin e6-Biotin Conjugates for Tumor-Targeting Photodynamic Therapy
Source: Molecules. 2021 Dec 3;26(23):7342. doi: 10.3390/molecules26237342 (PMC8658943; doi:10.3390/molecules26237342)
Supplement: Supplementary file 1 [file molecules-26-07342-s001.zip › molecules-1455728-supplementary.pdf]

Supplementary Material

Chlorin e6-Biotin Conjugates for Tumor-Targeting Photodynamic Therapy

Wei Liu [1,†], Xingqun Ma [2,†], Yingying Jin [1], Jie Zhang [1], Yang Li [1], Yuxia Tang [1,*], Yong Song [3,*] and Shouju Wang [1,*]

[1] Lab of Molecular Imaging, Department of Radiology, the First Affiliated Hospital of Nanjing Medical University, Nanjing 210000, China; njmu_lw@163.com (W.L.); yingyingjin@njmu.edu.cn (Y.J.);
zhangjiegxf@163.com (J.Z.); yang.li@njmu.edu.cn (Y.L.)

[2] Department of Oncology, Nanjing Baiyi Hospital, Jinling Clinical College of Nanjing Medical University, Nanjing 210000, China; maxingqun@126.com

[3] Department of Respiratory and Critical Care Medicine, Jinling Clinical College of Nanjing Medical University, Nanjing 210000, China

* Correspondence: tangyuxia5@163.com (Y.T.); yong_song6310@yahoo.com (Y.S.); shouju.wang@gmail.com (S.W.)

† These authors contributed equally

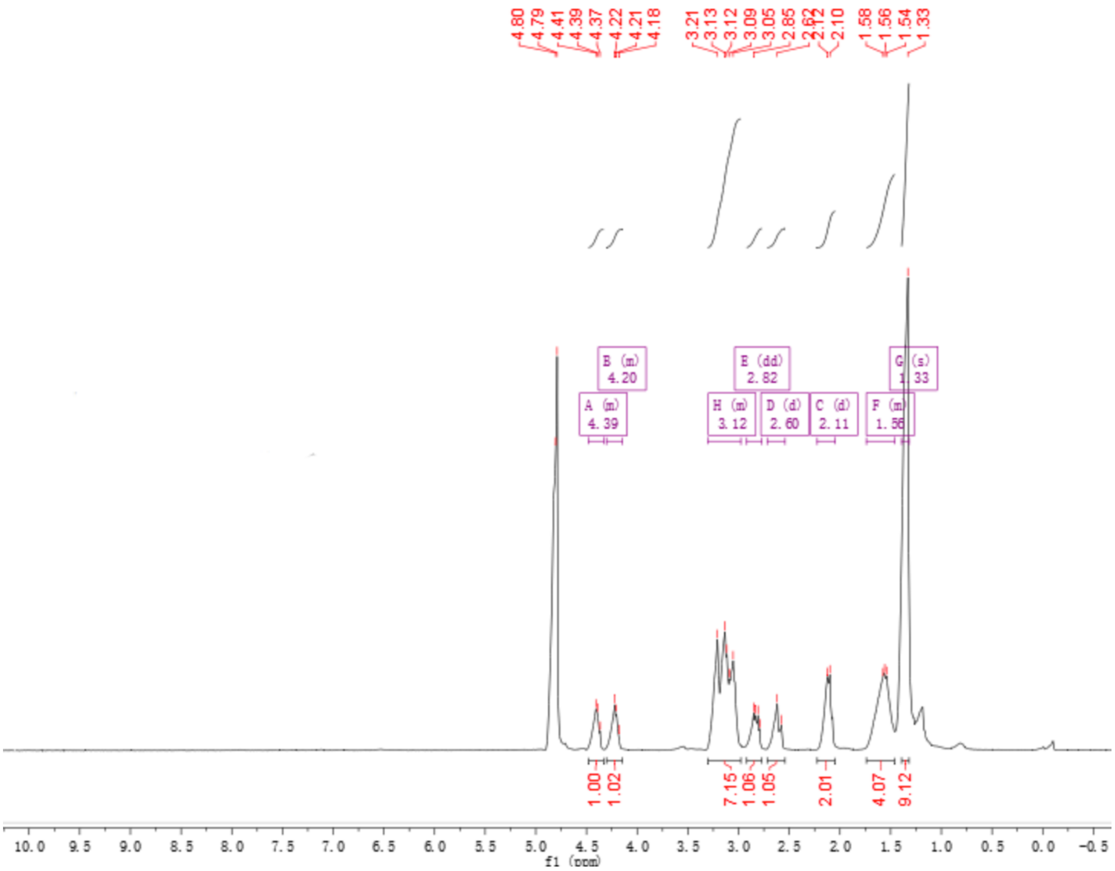

Figure S1. The ^{1}H-NMR of Boc-protected biotin (compound **2**).

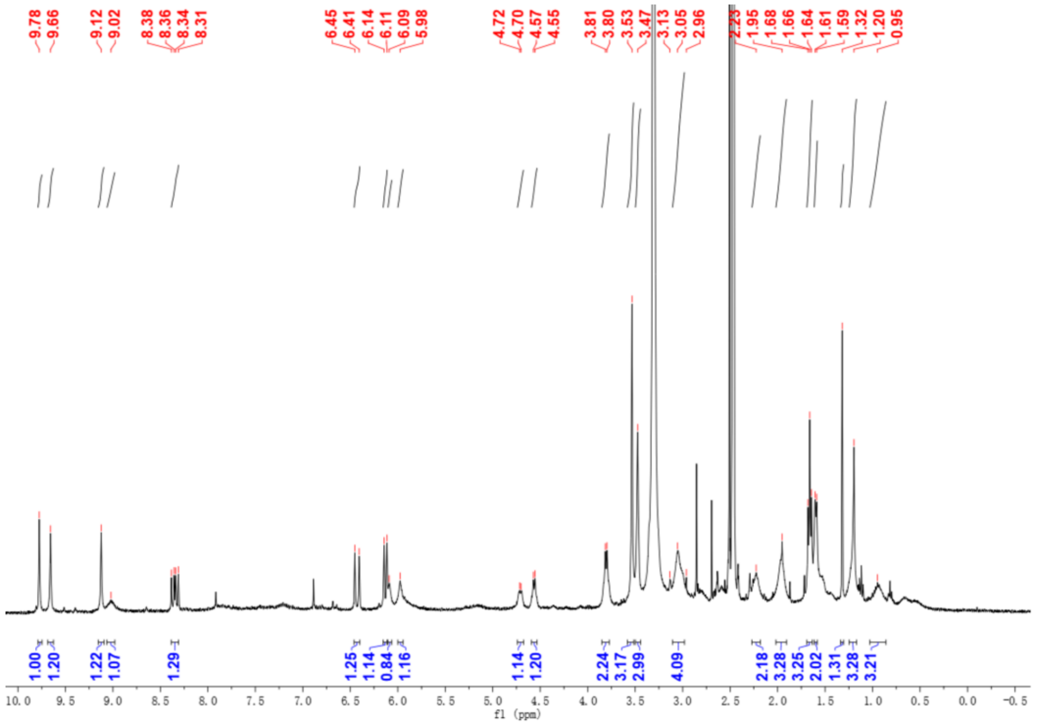

Figure S2. The ^{1}H-NMR of Ce6-biotin.

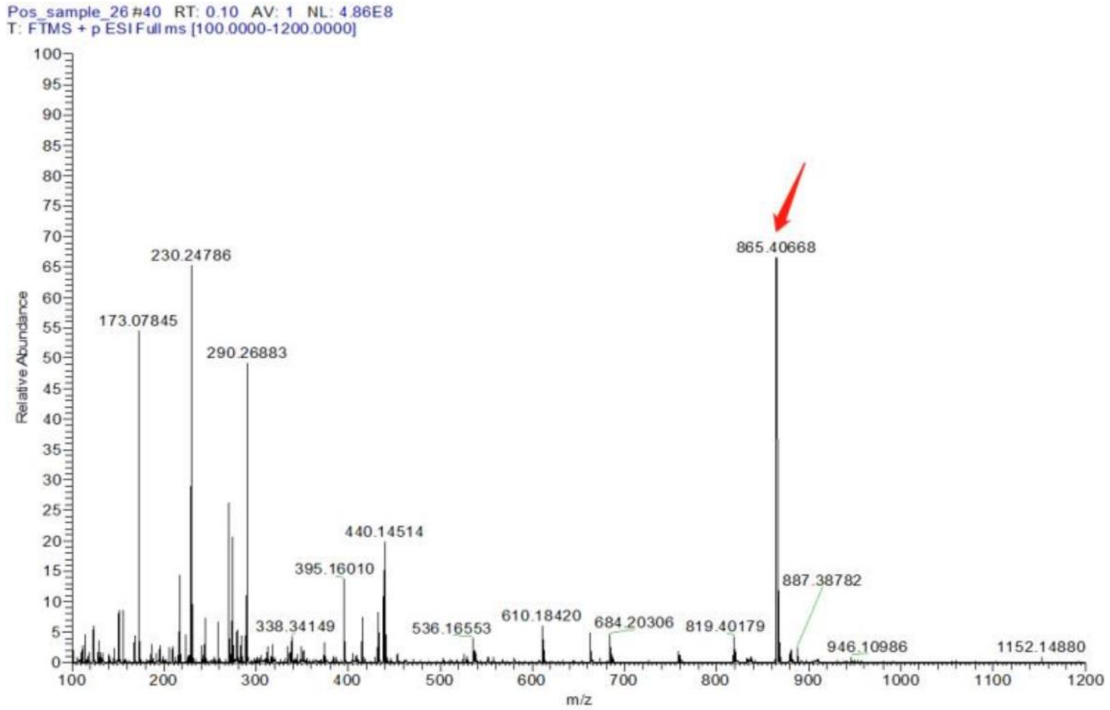

Figure S3. The HR-MS of Ce6-biotin.
